# Rambutan (*Nephelium lappaceum* L.) Shell as a Source of Polyphenols: Chemical Characterization and Biological Activities

**DOI:** 10.3390/molecules31111925

**Published:** 2026-06-03

**Authors:** Carlos Barba-Ostria, Arianna Mayorga-Ramos, Johana Zúñiga-Miranda, Rebeca Gonzalez-Pastor, Elena Coyago-Cruz, Antonella Viteri, Ana Belén Peñaherrera-Pazmiño, Orestes López, Diana Celi, Eduardo Tejera, Linda P. Guamán

**Affiliations:** 1 Escuela de Medicina, Colegio de Ciencias de la Salud Quito, Universidad San Francisco de Quito (USFQ), Quito 170901, Ecuador; 2 Instituto de Microbiología, Universidad San Francisco de Quito (USFQ), Quito 170901, Ecuador; 3 Facultad de Ciencias de la Salud Eugenio Espejo, Universidad UTE, Quito 170527, Ecuador; arianna.mayorga@ute.edu.ec (A.M.-R.); johana.zuniga@ute.edu.ec (J.Z.-M.); rebeca.gonzalez@ute.edu.ec (R.G.-P.); antonellaviteri28@gmail.com (A.V.); ana.penaherrera@ute.edu.ec (A.B.P.-P.); 4 Carrera de Ingeniería en Biotecnología, Universidad Politécnica Salesiana, Sede Quito, Campus El Girón, Av. 12 de Octubre N2422 y Wilson, Quito 170143, Ecuador; ecoyagoc@ups.edu.ec; 5 Facultad de Ciencia e Ingeniería en Alimentos y Biotecnología, Universidad Técnica de Ambato, Ambato 180104, Ecuador; od.lopez@uta.edu.ec; 6 Facultad de Ingeniería y Ciencias Aplicadas, Carrera de Ingeniería en Agroindustria, Universidad de Las Américas, Quito 170504, Ecuador; diana.celi@udla.edu.ec; 7 Bio-Cheminformatics Research Group, Universidad de Las Américas, Quito 170504, Ecuador; eduardo.tejera@udla.edu.ec; 8 Facultad de Ingeniería y Ciencias Aplicadas, Carrera Ingeniería en Biotecnología, Universidad de Las Américas, Quito 170504, Ecuador

**Keywords:** *Nephelium lappaceum*, ellagitannins, agro-industrial byproducts, antibiofilm activity, SDG 3, SDG 9

## Abstract

This study investigates the valorization of *Nephelium lappaceum* (rambutan) shell, an agro-industrial byproduct, as a sustainable source of bioactive compounds through comprehensive chemical and functional characterization. Phytochemical profiles were determined using spectrophotometrics and HPLC-DAD-MS/MS, revealing a composition dominated by ellagitannins (e.g., geraniin, corilagin, chebulagic acid) and ellagic acid derivatives, alongside significant levels of total phenolics (25,982.2 mg/100 g DW) and anthocyanins. The extract exhibited strong antioxidant activity (DPPH IC_50_ = 8.02 μg/mL; TEAC = 5703.92 μmol TE/g), consistent with its high phenolic content. Biological evaluation demonstrated antimicrobial activity against a broad panel of Gram-positive and Gram-negative bacteria, including multidrug-resistant strains, with greater efficacy against Gram-positive species (*Staphylococcus aureus*, MIC = 2.5 mg/mL). The extract also showed significant antibiofilm activity, achieving up to 93% inhibition. Antitumoral assays revealed selective cytotoxicity, particularly against HeLa cells (IC_50_ = 260 μg/mL; TI = 11.5), indicating preferential effects on tumor over non-tumor cells. Importantly, hemolytic assays confirmed low toxicity, with negligible erythrocyte membrane disruption across tested concentrations. Overall, these findings highlight rambutan shell as a rich source of phenolic bioactives with multifunctional biological properties and favorable safety profile, supporting its potential application in nutraceutical and pharmaceutical formulations within a circular economy framework. This study aligns with SDG 3 and SDG 9 by promoting the valorization of agro-industrial waste as a source of safe bioactive compounds for health-related applications.

## 1. Introduction

The increasing global burden of chronic diseases associated with oxidative stress, inflammation, and antimicrobial resistance has intensified the search for bioactive compounds derived from natural sources as alternatives to synthetic drugs and additives [1,2]. Among these, plant secondary metabolites, particularly polyphenols and anthocyanins, have attracted sustained scientific interest due to their broad spectrum of biological activities, including antioxidant, antimicrobial, anti-inflammatory, and antitumoral effects [3,4]. These compounds exert their functions through diverse mechanisms, such as modulation of redox homeostasis, disruption of microbial membranes, and interference with signaling pathways involved in cell proliferation and survival.

Agro-industrial byproducts are increasingly recognized as valuable reservoirs of phytochemicals, aligning with the goals of sustainable development and circular economy [5,6]. Fruit peels, seeds, and shells, often discarded as waste, are particularly rich in phenolic acids and flavonoids that can be exploited as functional ingredients [7].

Rambutan (*Nephelium lappaceum* L.), a tropical fruit widely consumed in Southeast Asia and increasingly available in global markets, represents a relevant example of such underutilized resources. Its shell, which constitutes a significant fraction of the fruit biomass, has been reported to contain high levels of phenolic compounds, including hydroxybenzoic and hydroxycinnamic acids, as well as anthocyanins responsible for its characteristic pigmentation [8,9,10]. More recently, studies have highlighted the presence of hydrolyzable tannins particularly ellagitannins such as geraniin, corilagin, and chebulagic acid which are recognized for their potent antioxidant and bioactive properties [10].

Previous investigations have demonstrated that rambutan shell extracts exhibit antioxidant and antimicrobial activities, with potential applications in food preservation and health-related contexts [9,10,11]. However, these studies have frequently relied on partial chemical characterization or have focused on single biological endpoints, limiting the understanding of how compositional complexity translates into multifunctional bioactivity. Furthermore, while individual compounds such as geraniin have been associated with specific biological effects [12], the contribution of the overall phytochemical matrix, particularly ellagitannin-rich fractions, remains insufficiently contextualized across different biological systems. In addition, critical aspects such as antibiofilm activity, tumor selectivity, and hemocompatibility have been comparatively less explored, despite their relevance for biomedical and translational applications.

In this context, the present study aims to provide an integrated evaluation of rambutan shell extract by combining chemical characterization (HPLC-DAD-MS/MS) with a broad panel of biological assays, including antioxidant, antibacterial (against both reference and multidrug-resistant strains), antibiofilm, antitumoral, and hemolytic activities. In addition, the present study establishes a distinctive biotechnological approach by deploying a targeted acidified extraction platform that preserves ellagitannins, while simultaneously corroborating late-eluting triterpenoid saponins and multi-targeted antibiofilm activities.

By linking compositional data with functional responses, this work seeks to better define the bioactive profile of this agro-industrial byproduct and to position it within a more mechanistically informed framework. This integrative approach contributes to advancing the valorization of *N. lappaceum* shell as a multifunctional source of bioactive compounds with potential applications in nutraceutical, pharmaceutical, and food systems, while aligning with circular economy strategies.

## 2. Materials and Methods

### 2.1. Plant Material

Ripe fruits of *Nephelium lappaceum* L. were purchased in December 2024 from the Wholesale Fruit Market of Ambato City, Ecuador. Fruits were selected at commercial maturity, corresponding to stage 6 of the visual ripening scale, characterized by an intense red coloration of the skin and spines. The shells were separated, washed, disinfected with a 100-ppm chlorine solution, and dried at 60 °C. The dried material was subsequently stored at room temperature. Prior to analysis, the samples were milled to obtain a fine powder, with particle sizes ranging from 100 to 500 μm, using an electric laboratory blender. The resulting powder was then passed through a No. 35 sieve with a 500-μm mesh size.

### 2.2. Bioactive Compounds Processing

Twenty milligrams of the sample were extracted with 2 mL of absolute ethanol, after which the sample was homogenised and sonicated for three minutes. The extract was then centrifuged at 14,000 rpm for five minutes at 4 °C and the resulting supernatant collected. Quantification was performed using the pH differential method in a 96-well microplate by mixing 50 µL of the extract with either 200 µL of potassium chloride (0.025 M, pH 1.0) or sodium acetate (0.4 M, pH 4.5) [13]. Absorbances were recorded at 520 and 700 nm using a microplate reader. Concentration was determined using a calibration curve with delphinidin chloride (1 mg/mL) and was expressed as mg of delphinidin chloride (D-ch) per 100 g of dry weight.

Quantification of ascorbic acid was carried out using liquid chromatography and *L*-ascorbic acid as the standard. A total of 20 mg of freeze-dried samples were weighed, extracted with 1.2 mL of 3% metaphosphoric acid and 200 µL of 0.2% homocysteine, and then homogenised and stirred for one minute in an ultrasonic bath FS60D (Fisher Scientific Inc., Waltham, MA, USA) at room temperature (25 °C), and 40 kHz. Then, 600 µL of deionised water was added, after which the solution was filtered using a 0.45 µm PDVF filter prior to analysis. Chromatographic analysis was performed using an RRLC 1200 system (Agilent, Santa Clara, CA, USA) coupled to a DAD-UV-Vis detector and a ZORBAX Eclipse XDB-C18 column (1.8 µm, 4.6 × 50 mm). The mobile phase was a mixture of 1.5% potassium monobasic phosphate in water (90%) and 1.8% cetyltrimethylammonium bromide in methanol (10%), at a flow rate of 1 mL/min. The injection volume was 20 µL, with detection performed at 244 nm. Quantification was performed using a calibration curve prepared from a 1 mg/mL standard solution of L-ascorbic acid with dilutions in the range 0.16–1.00 mg/mL (R^2^ > 0.99) [14].

To extract the phenolic compounds, 20 mg of the freeze-dried sample was weighed, treated with 500 µL of acidified methanol (80% with 0.1% HCl), then homogenised and sonicated for three minutes. This procedure was repeated three times and the resulting extracts were collected for analysis. Chromatographic separation was performed using an Agilent RRLC 1200 system (Santa Clara, CA, USA) with a DAD-UV-Vis detector and a ZORBAX Eclipse Plus C18 column (5 µm, 4.6 × 150 mm). The gradient mobile phase comprised 0.01% formic acid (A) and HPLC-grade acetonitrile (B), with a flow rate of 1 mL/min. The injection volume was 20 µL, with detection set to 280 nm for flavanones and 320 nm for hydroxycinnamic acids and flavones. Identification was carried out by comparing UV-Vis spectra and retention times with reference standards. Quantification was performed using individual calibration curves of phenolic acids derived from benzoic acid: gallic acid, protocatechuic acid, vanillic acid, syringic acid, p-hydroxybenzoic acid, 3-hydroxybenzoic acid, 2,5-dihydroxybenzoic acid, 2-methoxybenzoic acid and 3-methoxybenzoic acid; followed by hydroxycinnamic acids: caffeic acid, ferulic acid, chlorogenic acid, m-coumaric acid, p-coumaric acid and o-coumaric acid; as well as other compounds such as shikimic acid, ellagic acid and tannic acid (0.3–2.0 mg/mL; R^2^ > 0.99) [14].

The organic acids were extracted from 20 mg of dry extract using 1.5 mL of a solution comprising sulphuric acid (0.02 N), metaphosphoric acid (0.05%) and homocysteine (0.02%). The mixture was homogenised and sonicated for 3 min, followed by centrifugation at 14,000 rpm for 5 min at 4 °C, with the supernatant being recovered. The solid residue was re-extracted with 500 µL of the same solvent, and the combined extracts were filtered through a 0.45 µm PVDF membrane. Quantification was performed by HPLC coupled with a DAD-UV-Vis detector, using a YMC-Triart C18 column. Quantification was performed using individual calibration curves of citric, malic and tartaric acid (0.05–1.0 mg/mL; R^2^ > 0.99) [14].

### 2.3. Characterization by HPLC-DAD-MS/MS 

Acidified ethanolic extract was obtained following the procedure described by Perez et al. (2021) [15], with slight modifications. Briefly, the samples were combined with twenty volumes of a 96% ethanol–citric acid solution (85:15, m/m) and subjected to microwave-assisted extraction for 5 min. The resulting supernatant was separated using a Rotina 380 centrifuge (Hettich GmbH, Tuttlingen, Germany). The extract was then transferred into a 500 mL flask under constant stirring and concentrated with a rotary evaporator in a water bath at 68 °C under reduced pressure to eliminate the solvent [15].

HPLC-DAD-MS/MS analysis was performed using a Vanquish system (Thermo Fisher Scientific, Waltham, MA, USA) equipped with a dual pump and a diode array detector, and coupled to an LTQ-XL mass spectrometer (Thermo Fisher Scientific, Waltham, MA, USA). Instrument control and data acquisition were carried out with Xcalibur software (version 4.3).

Metabolite separation was achieved on a ZORBAX Eclipse Plus C18 column (4.6 × 150 mm, 5 μm; Agilent, Santa Clara, CA, USA) maintained at 35 °C. The mobile phase consisted of 0.1% formic acid in water (A) and acetonitrile (B). Elution was performed under gradient conditions as follows: 5% B from 0 to 5 min, 20% B from 5 to 10 min, 35% B from 10 to 20 min, 55% B from 20 to 30 min, 70% B from 30 to 35 min, and 90% B from 35 to 57 min, followed by re-equilibration at 5% B from 57 to 60 min. The flow rate was set at 0.2 mL/min and 50 μL was injected for each analysis.

UV absorbance was monitored at 220, 280, 330, and 370 nm. MS detection was carried out in negative electrospray ionization mode over an *m*/*z* range of 50–2000. The ion source was operated with a capillary temperature of 275 °C, source voltage of 4.5 kV, capillary voltage of 26 V, and tube lens voltage of 90 V.

Tentative metabolite identification was based on UV and MS/MS data, supported by comparison with the literature and relevant databases.

### 2.4. Antioxidant Capacity

The radical scavenging capacity of the acidified ethanolic extract of *Nephelium lappaceum* L. was assessed using the DPPH radical scavenging assay and the ABTS radical-cation decolorization assay. For these analyses, 1.2 mg of extract was dissolved in 1.2 mL of DMSO to obtain a final concentration of 1 mg/mL. Ascorbic acid was used as the reference antioxidant, and a 200 μg/mL stock solution was prepared in Milli-Q water.

The DPPH microplate assay was performed according to a protocol adapted from [16]. Briefly, stock solutions of the extract and standard were prepared at 100 μg/mL and subjected to two-fold serial dilutions in methanol, reaching a final volume of 200 μL per well. The reaction mixtures were incubated for 45 min in the dark at room temperature, after which absorbance was measured at 515 nm using a Cytation 5 plate reader (BioTek, Malvern, PA USA, Malvern, PA USA).

DPPH radical scavenging activity was calculated using the following equation:


$$DPPH\;scavenging\;\%=100\;\times \left[\left(A_{sample}+\;DPPH-A_{sample\;blank}\right)/\left(A_{DPPH}-A_{solvent}\right)\right]\;$$

The antioxidant activity of the *N. lappaceum* acidified ethanolic extract was further expressed as the half-maximal inhibitory concentration (IC_50_), defined as the concentration required to inhibit 50% of radical activity. IC_50_ values were obtained by plotting the percentage of inhibition against the corresponding sample concentrations. From the resulting linear regression equation, y = mx + c, the x-value corresponding to y = 50 was calculated and reported as the IC_50_.

The ABTS decolorization assay was conducted following a procedure adapted from the literature [16]. Briefly, a 7 mM ABTS solution was mixed with ammonium persulfate (APS) to obtain a final APS concentration of 2.45 mM. The mixture was maintained in the dark for 16 h to allow formation of the ABTS radical cation. Before use, the ABTS radical solution was diluted with water until an absorbance of approximately 0.7 at 734 nm was reached.

A 2 mM Trolox stock solution was prepared in the PBS buffer at pH 7.2 and subsequently diluted to obtain standard solutions ranging from 12.5 to 400 μM. These solutions were used to construct a calibration curve for determining the Trolox equivalent antioxidant capacity (TEAC) of the samples. The Trolox concentrations that produced the same percentage decrease in absorbance at 734 nm as the extract and ascorbic acid were calculated, and the results were expressed as μmol Trolox equivalents per gram (μmol TE/g).

For the ABTS assay, stock solutions of *N. lappaceum* acidified ethanolic extract and ascorbic acid were prepared at concentrations of 100 μg/mL and 50 μg/mL, respectively. The extract was diluted in DMSO, whereas ascorbic acid was diluted in water, using two-fold serial dilutions. Subsequently, 10 μL of each dilution was mixed with 190 μL of ABTS radical solution in a 96-well microplate. The reaction mixtures were incubated for 5 min in the dark at room temperature, and absorbance was recorded at 734 nm using a Cytation 5 plate reader (BioTek, Malvern, PA USA, Malvern, PA USA).

The percentage of ABTS decolorization was calculated as follows:


$$Decolorization\;\%\;=\;100\times \left[\left(A_{solvent}-A_{sample}\right)/\left(A_{solvent}\right)\right]$$

Antioxidant capacity relative to Trolox was determined from the calibration-curve equation using the following expression:

$$Trolox-eq\left(\frac{\mu mol}{g}\right)=\left[\frac{\left(Sample\;decolorization\;(\%\right)-b)}{a}\right]/\;Sample\;concentration\;(mg/L)$$

where a and b correspond to the slope and intercept of the *Trolox* calibration curve, respectively.

Antioxidant activity was expressed as IC_50_ values, representing the concentration required to scavenge 50% of DPPH radicals or to decolorize 50% of the ABTS radical solution. IC_50_ values were calculated using GraphPad Prism version 10.4.2 (GraphPad Software, Boston, MA, USA). All assays were performed in triplicate, and results are reported as mean ± standard deviation (SD).

### 2.5. Antibacterial Evaluation

The antibacterial activity of *N. lappaceum* acidified ethanolic extract was assessed against seven non-multidrug-resistant bacteria, including Gram-negative strains *Salmonella enterica* ATCC 14028, *Escherichia coli* ATCC 25922, *Pseudomonas aeruginosa* ATCC 27853, and *Burkholderia cepacia* ATCC 25416. Gram-positive strains: *Staphylococcus aureus* ATCC 25923, *Enterococcus faecalis* ATCC 29212, and *Listeria monocytogenes* ATCC 13932. And seven multidrug-resistant bacteria provided by the National Health Institute of Ecuador (INSPI): *Klebsiella pneumoniae*, *Salmonella enterica* serovar Kentucky, *Staphylococcus epidermidis*, *Enterococcus faecium*, and *Pseudomonas aeruginosa*.

The minimal inhibitory concentration (MIC) was determined using the microdilution method, as outlined in the Clinical and Laboratory Standards Institute (CLSI) guidelines [17]. The *N. lappaceum* extract was serially diluted in DMSO; then, 5 μL of each dilution was added to 195 μL of a bacterial suspension (5 × 10^5^ CFU/mL) to a total volume of 200 μL. The final concentration of *N. lappaceum* extract in each well ranged from 0.31 to 20 mg/mL. As a control, bacterial cells were grown with DMSO. Additionally, several antibiotics were used as controls for growth inhibition at the recommended working concentrations for the tested strains (Table 1). Both BHI alone and supplemented with *N. lappaceum* extract at different concentrations were used as blanks.

### 2.6. Biofilm Inhibition Evaluation

The antibiofilm activity of *N. lappaceum* extract was evaluated against four biofilm-forming bacterial strains *S. aureus* ATCC 25923, *L. monocytogenes* ATCC 13932, *Pseudomonas aeruginosa* ATCC 9027, and *Burkholderia cepacia* ATCC 25416 using a crystal violet microtiter plate assay as described previously by Barba-Ostria et al. [18]. The extract was evaluated in serial concentrations ranging between 10 mg/mL and 1 µg/mL and the MBIC_50_ (Minimum Biofilm Inhibitory Concentration for ≥50% reduction in biofilm formation) was determined.

### 2.7. Antitumoral Evaluation

The antitumoral activity of *N. lappaceum* extract was evaluated through in vitro proliferation assays using a panel of human cancer cell lines and a non-tumoral cell line to assess selectivity. Human tumor cell lines HeLa (cervical carcinoma, RRID:CVCL_0030), THJ29T (thyroid carcinoma, Cat. No. T8254, RRID:CVCL_W922), HepG2 (hepatocellular carcinoma, ATCC: HB-8065, RRID:CVCL_0027), and HCT116 (colorectal carcinoma, RRID:CVCL_0291), along with the non-tumor NIH3T3 cell line (mouse NIH/Swiss embryo fibroblasts, ATCC: CRL-1658, RRID:CVCL_0594), were cultured in Dulbecco’s Modified Eagle Medium/Nutrient Mixture F-12 (DMEM/F12; Corning, NY, USA) supplemented with 10% fetal bovine serum (FBS; Capricorn Scientific, Ebsdorfergrund, Dreihausen, Germany) and 1% penicillin–streptomycin (Gibco, Thermo Fisher Scientific, Waltham, MA, USA). All cell lines were maintained at 37 °C in a humidified atmosphere containing 5% CO_2_.

For MTT assay, cells were seeded in 96-well plates and allowed to adhere for 24 h prior to treatment. HeLa, THJ29T, and NIH3T3 cells were plated at a density of 1 × 10^4^ cells/well in 100 μL of complete medium, whereas HepG2 and HCT116 were seeded at densities of 2 × 10^4^ and 1.5 × 10^4^ cells/well, respectively, according to their growth characteristics. The extract was prepared as a stock solution (500 mg/mL) in DMSO, stored at 4 °C, and protected from light to preserve metabolite stability. Working solutions were prepared by serial two-fold dilution in complete medium to obtain final concentrations ranging from 0.08 to 5 mg/mL. The final concentration of DMSO did not exceed 1% (*v*/*v*) at any tested concentration. Untreated cells were used as negative controls. Cells were exposed to the extract for 72 h under standard culture conditions. Cell proliferation was subsequently assessed using the MTT assay (3-(4,5-dimethylthiazol-2-yl)-2,5-diphenyltetrazolium bromide; Sigma-Aldrich, St. Louis, MO, USA), which is based on the reduction of tetrazolium salts to insoluble formazan crystals by metabolically active cells. Briefly, 10 μL of MTT solution (5 mg/mL in PBS) was added to each well and incubated for 1 h at 37 °C. The medium was then carefully removed, and the resulting formazan crystals were solubilized in 50 μL of DMSO. Plates were gently agitated (300 rpm, 1 min) before measuring absorbance at 570 nm using a Cytation 5 multi-mode reader (BioTek, Malvern, PA, USA). Cell proliferation was expressed as a percentage relative to untreated control cells, which were considered as 100% proliferation. All treatments were performed in quadruplicate, and experiments were repeated in at least three independent biological replicates to ensure reproducibility. Dose–response curves were generated using nonlinear regression (four-parameter logistic model) in GraphPad Prism 9.4.1 software (GraphPad Software, Boston, MA, USA), and the half-maximal inhibitory concentration (IC_50_) was calculated accordingly. The therapeutic index (TI) was calculated as the ratio between the IC_50_ value obtained in the non-tumoral cell line (NIH3T3) and that of each tumor cell line.

### 2.8. Hemolytic Assay

The hemolytic activity of the *N. lappaceum* shell extract was determined as previously described [19]. Briefly, 10 mL of defibrinated sheep blood was washed three times with 1× PBS, and the resulting erythrocytes were resuspended in 1× PBS to obtain a 1% suspension. Sheep blood was obtained from Ayllu Ovis (Quito, Ecuador) that follows stringent protocols for safe blood collection and processing, ensuring compliance with biosafety and ethical guidelines. This suspension was then mixed at a 1:1 ratio with *N. lappaceum* shell extract at the indicated concentrations, with 10% Triton X-100 as a positive control and 2.5% DMSO as a negative control, in a 96-well polypropylene plate. After incubation at 37 °C for 1 h, the samples were centrifuged at 1700× *g* for 5 min. The supernatants were carefully transferred to a transparent flat-bottom 96-well plate, and absorbance was measured at 405 nm using a Cytation 5 multimode plate reader (BioTek, Malvern, PA, USA). Each experiment was performed with three technical replicates and independently repeated three times. Hemolytic activity was interpreted according to the thresholds defined in the ASTM E2524-22 standard [20], ensuring alignment with contemporary hemocompatibility criteria.

### 2.9. Statistical Analysis

Statistical evaluation of biofilm inhibition was carried out using a two-way ANOVA followed by Dunnett’s post hoc test. Data from three independent biological replicates were included in the analysis. All analyses and graphical representations were generated using GraphPad Prism 10.2 (GraphPad Software, Boston, MA, USA). Differences were considered statistically significant at *p* values < 0.05, <0.01, and <0.001.

## 3. Results and Discussion

### 3.1. Bioactive Compounds

To evaluate the recovery efficiency of the agro-industrial byproduct valorization, the extraction yield was determined. The optimized microwave-assisted extraction (MAE) process operating at 1200 W for 5 min demonstrated high efficiency, achieving a global extraction yield of 30.03% ± 0.47% (*w*/*w*) from the *N. lappaceum* shell matrix.

The quantitative analysis of bioactive compounds in *N. lappaceum* shell extract (Table 2) revealed an exceptionally high total phenolic content (25,982.2 mg/100 gDW), positioning this matrix among the most phenolic-rich agro-industrial byproducts reported to date. This value exceeds those described for several tropical fruit residues, reinforcing the relevance of rambutan shell as a concentrated source of redox-active metabolites [21].

Within the phenolic profile, the predominance of hydroxycinnamic acids, particularly *m*-coumaric acid (Table 2), suggests a phytochemical composition shaped by plant defense mechanisms against environmental stressors such as UV radiation and oxidative pressure [22]. These compounds are known to contribute to antioxidant activity through electron donation and radical stabilization, as well as to antimicrobial effects via interactions with microbial membranes and intracellular targets [23,24].

Anthocyanin content, although comparatively lower than total phenolics, reflects the contribution of pigmented flavonoids to the overall bioactivity of the extract. Their concentration is influenced by intrinsic (species, tissue type) and extrinsic (processing conditions, temperature, oxidation) factors, which can affect their stability and bioavailability [25]. The organic acid profile was dominated by malic acid, consistent with its central role in plant metabolism, particularly within the tricarboxylic acid cycle, and its contribution to osmotic balance and fruit acidity [26]. While organic acids are not the primary contributors to bioactivity, their presence may modulate extract stability, pH-dependent solubility, and potential synergistic effects with phenolic compounds.

Taken together, these results define *N. lappaceum* shell extract as a phenolic-dense matrix, with a chemical composition dominated by hydroxycinnamic acids and supported by anthocyanins and organic acids. This compositional framework provides a biochemical basis for the multifunctional biological activities observed in subsequent assays.

### 3.2. HPLC-DAD-MS/MS

**Figure 1:**
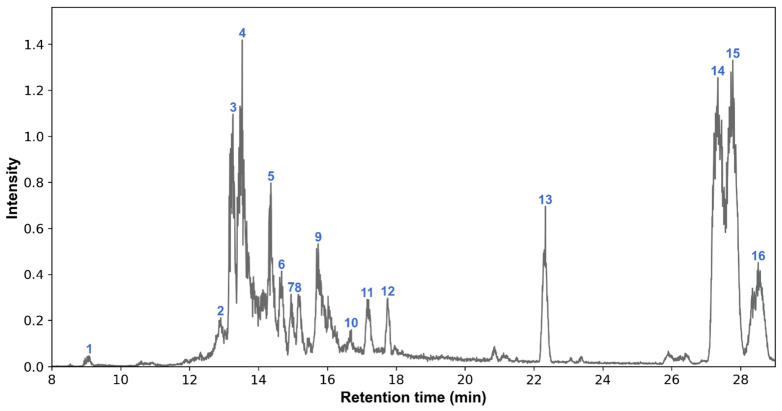
Chromatographic profile of *N. lappaceum* extract analyzed by HPLC-DAD-MS/MS in negative ionization mode. Peak numbers (1–16) correspond to the compounds identified and listed in Table 3.

Microwave-assisted extraction (MAE) platform at 1200 W for 5 min yielded an extraction recovery of 30.03% ± 0.47% on a dry weight basis (*w*/*w*). HPLC-DAD-MS/MS analysis revealed that the phytochemical signature of *N. lappaceum* shell extract is dominated by hydrolyzable tannins, particularly ellagitannins and ellagic acid derivatives, which constitute the major fraction of resolved metabolites (Table 3; Figure 1). This profile is consistent with previous reports describing geraniin, corilagin, and ellagic acid as principal constituents of rambutan shell [10].

The early chromatographic region was characterized by ions corresponding to galloyl derivatives and ellagitannins. For instance, ID1 (*m*/*z* 331) was tentatively assigned to galloylglucose, representing a fundamental structural unit in hydrolyzable tannin biosynthesis. Similarly, ID2 (*m*/*z* 631) was consistent with ellagitannin-related structures such as castalagin/vescalagin-type frameworks, which are commonly detected in tannin-rich plant matrices.

More structurally resolved assignments were achieved for IDs 3–6. Corilagin was tentatively proposed for ID3 (*m*/*z* 633) based on dominant fragments at *m*/*z* 301 (ellagic acid), 463 (loss of gallic acid), and 615 (dehydration) [27,28]. ID4 (*m*/*z* 951) displayed fragmentation consistent with geraniin, showing dehydration at *m*/*z* 933 and the diagnostic *m*/*z* 301, while ID5 (*m*/*z* 953) was tentatively identified as chebulagic acid, also exhibiting *m*/*z* 301 [29]. Their elution order matches previous reports [27,30], and both compounds have been documented in *N. lappaceum* [31,32]. A castalagin-type derivative was tentatively assigned to ID6 (*m*/*z* 965), supported by fragments at *m*/*z* 933 (−18 Da), 915, 903, and 301, consistent with this ellagitannin class [33,34,35].

ID7 (*m*/*z* 433) was tentatively characterized as an ellagic acid pentoside based on loss of a pentose (−132 Da) yielding *m*/*z* 301, whose identity was confirmed by characteristic fragments at *m*/*z* 257, 229, and 185 [36,37]. IDs 9–11 (*m*/*z* 997, 979, and 923) did not match known patterns but consistently displayed *m*/*z* 301, supporting their tentative classification as ellagitannins [28].

IDs 9–11 (*m*/*z* 997, 979, and 923) did not match any known fragmentation patterns in available databases or literature. However, all three displayed an intense signal at *m*/*z* 301, consistent with the ellagic acid, together with the characteristic secondary fragmentation cascade at *m*/*z* 257, 229, and 185. Given the confirmed diagnostic value of this ion series for ellagitannin class assignment, these compounds are tentatively classified as ellagitannins.

The late-eluting region was characterized by triterpenoid saponins. IDs 14 and 15 (*m*/*z* 882) showed sequential losses of 132, 146, and 132 Da (pentose–deoxyhexose–pentose sequence), yielding the hederagenin aglycone at *m*/*z* 472 with fragments at *m*/*z* 394 (base peak), 405, 427, and 440. This assignment was supported by a GNPS cosine score of 0.886 and importantly, this compound has been previously recorded in the *Nephelium* genus in both the COCONUT [38] and LOTUS [39] databases. ID16 was tentatively identified as α-hederin based on losses of a deoxyhexose and pentose leading to the same aglycone at *m*/*z* 472 [40].

**Table 3:** Tentative identification of phytochemical compounds in *Nephelium lappaceum* shell extract by HPLC-DAD-MS/MS in negative ionization mode.

ID	RT	*m*/*z* [M-H]^−^	MS/MS	Tentative Identification	Resource
1	9.09	311	125(10) 169(80) 271(100) 313(15)	Galloyglucose	[27,35]
2	12.90	631	445(19) 461(19) 613(100)	Ellagitannin related to castalagin/vescalagina	[41]
3	13.26	633	275(15) 301(100) 463(27) 615(24)	Corilagin	[27,28]
4	13.52	951	301(5) 613(5) 914(10) 933(100)	Geraniin	[27]
5	14.37	953	301(30) 425(10) 633(10) 934(100)	Chebulagic acid	[29]
6	14.67	965	301(10) 443(20) 903(40) 915(35) 933(100) 947(15)	Castalagin derivative	[33,34,35]
7	14.94	433	300(70) 301(100) 301 → 185(10) 229(50) 257(100) 273(5)	Ellagic acid pentoside	[42]
8	15.15	497	451(100)	UI	
9	15.73	997	301(80) 319(30) 435(25) 481(40) 827(15) 907(65) 952(100) 980(50) 923(45) 301 → 185(10) 229(50) 257(100) 273(5)	A type of Ellagitannin	^a^
10	16.68	979	301(80) 463(25) 631(10) 765(10) 809(100) 934(50) 960(30) 301 → 185(10) 229(50) 257(100) 273(5)	A type of Ellagitannin	^a^
11	17.15	923	301(100) 603(50) 621(40) 707(25) 906(35) 301 → 185(10) 229(50) 257(100) 273(5)	A type of Ellagitannin	^a^
12	17.75	1000	319(10) 525(15) 853(100) 953(30) 853 → 367(100) 707(40) 545(10)	UI	
13	22.33	984	367(30) 465(20) 513(10) 619(50) 837(100) 837 → 367(100) 691(50) 513(5) 367 → 221(100) 161(30) 205(20) 131(15)	UI	
14	27.72	882	472(35) 604(100) 733(25) 750(45) 472 → 394(100) 405(30) 440(25) 427(10)	10-[3-[3,5-dihydroxy-6-methyl-4-(3,4,5-trihydroxyoxan-2-yl)oxyoxan-2-yl]oxy-4,5-dihydroxyoxan-2-yl]oxy-9-(hydroxymethyl)-2,2,6*a*,6*b*,9,12*a*-hexamethyl-1,3,4,5,6,6*a*,7,8,8*a*,10,11,12,13,14*b*-tetradecahydropicene-4*a*-carboxylic acid (Isomer 1)	GNPS 0.886
15	27.77	882	472(35) 604(100) 733(25) 750(45)	10-[3-[3,5-dihydroxy-6-methyl-4-(3,4,5-trihydroxyoxan-2-yl)oxyoxan-2-yl]oxy-4,5-dihydroxyoxan-2-yl]oxy-9-(hydroxymethyl)-2,2,6*a*,6*b*,9,12*a*-hexamethyl-1,3,4,5,6,6*a*,7,8,8*a*,10,11,12,13,14*b*-tetradecahydropicene-4*a*-carboxylic acid (Isomer 2)	GNPS 0.857
16	28.51	750	472(100) 424(10) 586(95) 604(95)	α-hederin	[27]

^a^ No specific literature match available. Tentatively classified as ellagitannins based on the diagnostic reporter ion at *m*/*z* 301 and secondary fragmentation at *m*/*z* 257, 229, and 185. See Section 3.2 for details.

The late-eluting region was characterized by triterpenoid saponins. IDs 14 and 15 (*m*/*z* 882) showed sequential neutral losses of 132, 146, and 132 Da, consistent with a pentose–deoxyhexose–pentose sugar release sequence, yielding the hederagenin aglycone at *m*/*z* 472. MS^3^ fragmentation of this aglycone ion produced characteristic fragments at *m*/*z* 394 (base peak), 405, 427, and 440, corresponding to sequential dehydration and decarboxylation losses from the hederagenin skeleton, consistent with previously reported fragmentation data [DOI: 10.1039/C4AY02837F]. Accordingly, IDs 14 and 15 are tentatively assigned as two isomers of 10-[3-[3,5-dihydroxy-6-methyl-4-(3,4,5-trihydroxyoxan-2-yl)oxyoxan-2-yl]oxy-4,5-dihydroxyoxan-2-yl]oxy-9-(hydroxymethyl)-2,2,6a,6b,9,12a-hexamethyl-1,3,4,5,6,6a,7,8,8a,10,11,12,13,14b-tetradecahydropicene-4a-carboxylic acid. This assignment is further supported by a GNPS cosine score of 0.886, based on 19 matched fragment ions between the experimental spectrum and the library reference, and by the previously documented occurrence of this compound in the Nephelium genus in both the COCONUT [39] and LOTUS [40] databases. ID16 was tentatively identified as α-hederin based on losses of a deoxyhexose and pentose leading to the same aglycone at *m*/*z* 472 [40].

Notably, several ions (IDs 8, 12, and 13) remained unresolved despite multistage fragmentation analysis, indicating the presence of medium- to high-mass constituents that could not be confidently assigned based on available spectral data. This highlights the intrinsic complexity of the extract and suggests that additional approaches, such as high-resolution MS databases or NMR-based structural elucidation, may be required for full characterization. It is also important to notice that we did not identify any anthocyanin compound. We also used positive ionization mode in order to specifically explore anthocyanins, however none was identified in positive or negative ionization mode.

From a functional perspective, the predominance of ellagitannins provides a plausible chemical basis for the observed antioxidant and antimicrobial activities, as these compounds are known to exhibit strong radical scavenging capacity, protein-binding properties, and membrane-interactive behavior. In addition, the presence of saponins may contribute to bioactivity through surfactant-like interactions with biological membranes, potentially enhancing permeability and synergizing with phenolic constituents [43,44].

Overall, the HPLC-DAD-MS/MS analysis establishes *N. lappaceum* shell extract as a chemically complex system dominated by ellagitannin-type polyphenols, with secondary contributions from saponins and unresolved metabolites. This compositional profile supports a multifunctional bioactivity pattern and underscores the importance of considering the extract as an integrated phytochemical matrix rather than a sum of isolated compounds.

### 3.3. Antioxidant Activity

The antioxidant capacity of *N. lappaceum* shell extract was evaluated using DPPH and ABTS radical scavenging assays, together with TEAC quantification (Table 4). The extract exhibited a DPPH IC_50_ value of 8.02 μg/mL, indicating strong radical scavenging activity according to established classification criteria [45,46]. Although ascorbic acid displayed higher activity, the extract’s performance remains within the range reported for potent plant-derived antioxidants.

This activity is consistent with previous studies on rambutan shell extracts, which reported IC_50_ values ranging from 4.94 to 26.22 μg/mL [47,48], and supports the role of phenolic compounds as the primary contributors to antioxidant capacity. In particular, ellagitannins such as geraniin and corilagin have been identified as key redox-active molecules due to their multiple hydroxyl groups, which enable efficient electron donation and radical stabilization [10].

The higher IC_50_ observed in the ABTS assay compared to DPPH suggests differences in radical accessibility and reaction mechanisms, as ABTS is more sensitive to both hydrophilic and lipophilic antioxidants, whereas DPPH primarily reflects hydrophobic interactions [49]. The high TEAC value (5703.92 μmol TE/g) further supports the extract’s substantial antioxidant potential.

From a mechanistic perspective, the antioxidant activity of the extract can be attributed to the combined effects of phenolic hydroxyl groups, resonance stabilization of phenoxyl radicals, and possible metal-chelating properties, which together contribute to the modulation of oxidative processes [25]. These properties are particularly relevant in biological systems where oxidative stress plays a central role in disease development.

### 3.4. Antibacterial Activity

The *N. lappaceum* L. shell extract exhibited broad-spectrum antibacterial activity against 13 bacterial strains, including both multidrug-resistant (MDR) and non-MDR pathogens, with minimum inhibitory concentration (MIC) values ranging from 2.5 to 20 mg/mL. Overall, Gram-positive bacteria showed higher susceptibility (mean MIC: 7.9 mg/mL) compared to Gram-negative bacteria (mean MIC: 14.3 mg/mL). This differential response is consistent with the well-established structural differences in bacterial cell envelopes, as Gram-positive bacteria lack the outer membrane barrier characteristic of Gram-negative species [50,51].

In contrast, Gram-negative bacteria displayed higher MIC values (range: 10–20 mg/mL), which can be attributed to the protective role of the outer membrane lipopolysaccharide layer that limits the penetration of antimicrobial compounds. Although the observed MIC values indicate moderate antibacterial activity when compared to conventional antibiotics, they are consistent with those reported for plant-derived extracts and support potential applications in non-systemic contexts, such as food preservation or topical formulations [52]. Importantly, the extract retained activity against MDR strains, suggesting that its mechanism of action may differ from that of conventional antibiotics and could involve multi-target interactions that reduce the likelihood of resistance development Table 5 [52,53].

Phenolic compounds, particularly ellagitannins, are known to exert antimicrobial effects through several mechanisms, including membrane destabilization, protein precipitation, enzyme inhibition, and interference with microbial metabolism [54]. The high phenolic content of the extract (25,982.2 mg/100 g DW) supports its role as the primary driver of antibacterial activity.

A notable finding of this study is the extract’s effectiveness against six MDR clinical isolates. The MIC values for MDR strains (10–20 mg/mL) were only slightly higher than those observed for non-MDR reference strains, with no evidence of complete resistance. This pattern suggests that the polyphenolic constituents may act through mechanisms distinct from those of conventional antibiotics, potentially bypassing common resistance pathways such as efflux pumps, β-lactamase activity, or target-site modifications [52,53].

Particularly relevant is the comparable activity observed against MDR and non-MDR enterococcal strains (both 10 mg/mL), given that vancomycin-resistant enterococci (VRE) represent a significant clinical challenge. Furthermore, the extract demonstrated activity against MDR *Pseudomonas aeruginosa* and *Klebsiella pneumoniae*, both of which are classified as priority pathogens by the World Health Organization. Overall, while the antibacterial potency is moderate, the broad-spectrum activity and effectiveness against MDR strains highlight the potential of *N. lappaceum* shell extract as a natural antimicrobial agent, particularly in applications where high concentrations can be locally achieved.

### 3.5. Biofilm Inhibition Activity

**Figure 2:**
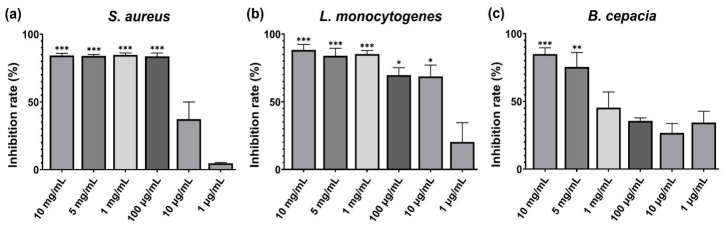
Biofilm inhibition activity of *N. lappaceum* shell extract against (**a**) *Staphylococcus aureus*, (**b**) *Listeria monocytogenes*, and (**c**) *Burkholderia cepacia*. Treatments at different concentrations were compared with a 50% theoretical inhibition control for statistical significance using a two-way ANOVA test. Bars represent mean ± standard deviation (*n* = 3), *p*-value (*) < 0.05, (**) < 0.01, and (***) < 0.001.

Evaluation of the biofilm inhibition potential (MBIC_50_) of *N. lappaceum* shell extract exhibited significant inhibitory effects on biofilm formation by *S. aureus* and *L. monocytogenes* achieving an MBIC_50_ of 84% ± 2.57% (*p* ≤ 0.001) at 100 µg/mL and 85% ± 2.45% (*p* ≤ 0.001) at 1 mg/mL, respectively (Figure 2). The extract also displayed significant inhibition activity against *B. cepacia* at 5 mg/mL (75% ± 10.6%, *p* ≤ 0.01). In contrast, no statistically significant inhibition was observed against *P. aeruginosa* at any of the tested concentrations.

Biofilm formation is a complex, multi-step process involving cell adhesion, extracellular matrix production, and quorum sensing regulation. The observed antibiofilm activity suggests that the extract interferes with one or more of these processes, potentially through disruption of cell surface interactions or inhibition of signaling pathways involved in biofilm development.

Polyphenols have been reported to inhibit biofilm formation by altering membrane properties, reducing extracellular polymeric substance (EPS) production, and modulating quorum sensing systems [52,53,55]. In this context, ellagitannins may play a central role due to their ability to interact with proteins and polysaccharides, thereby affecting biofilm structural integrity.

In one of our previous studies, the effects of microencapsulated *N. lappaceum* were evaluated, and the microencapsulated extract demonstrated significant activity against *S. aureus* and *L. monocytogenes* [18]. In this regard, the present study revealed that the crude *N. lappaceum* extract exhibited similar inhibition rates against these bacteria comparable to those of its microencapsulated counterpart. Additionally, no differences were observed in the MBIC_50_ recorded for these strains.

Notably, the crude extract did exhibit some activity against *B. cepacia*, an effect that had not been previously detected for the microencapsulated extract [18]. Some studies have suggested that microencapsulated extracts can exhibit lower efficacy than crude extracts against certain biofilm-forming strains, particularly in reducing key biofilm-associated features such as the extracellular polymeric substance (EPS) matrix [56]. This reduced activity has been observed in relation to major EPS components, including proteins and polysaccharides, which play essential roles in biofilm structural integrity, adhesion, and antimicrobial tolerance [56].

Our extract did not display any effect against *P. aeruginosa* biofilm, this may be related to some of its intrinsic resistance mechanisms, including robust efflux systems and adaptive biofilm architecture [57]. The mechanisms by which *P. aeruginosa* biofilms exhibit antimicrobial resistance has been extensively documented in the literature [57], which may also explain the differential activity observed for our extract against this bacterium compared with *S. aureus* and *L. monocytogenes*. This highlights the importance of species-specific responses and suggests that the efficacy of plant-derived extracts in biofilm control may depend on the target organism.

### 3.6. Antitumoral Activity

The antiproliferative activity of *N. lappaceum* extract was evaluated across a panel of human tumor cell lines, revealing IC_50_ values ranging from 260 to 3086 μg/mL (Table 6). Among these, HeLa cells were the most sensitive (IC_50_ = 260 μg/mL), while THJ29T cells exhibited the highest resistance.

The *N. lappaceum* extract exhibited IC_50_ values ranging from 260 to 2678 µg/mL across the evaluated cell lines. Among them, HeLa cells were the most sensitive, showing the lowest IC_50_ value (260 µg/mL), and THJ29T cells were the least sensitive, presenting the highest IC_50_ value (3086 µg/mL). Regarding selectivity, the extract showed TI values above 2 for HepG2, HCT116, and HeLa cells, with the highest TI observed in HeLa cells (TI = 11.5). This value indicates strong selectivity toward this tumor cell line. By contrast, THJ29T showed a TI below 1, indicating no selectivity in this cell line. Although cisplatin (CDDP) displayed significantly lower IC_50_ values in all tested cell lines, its therapeutic index values were comparatively lower, indicating reduced selectivity relative to the extract. Notably, most TI values obtained for the extract were above 1, with some exceeding 2, supporting a preferential cytotoxic effect on tumor cells over non-tumoral cells. These findings suggest that the extract may exert a more selective antiproliferative effect in hepatocellular, colorectal, and cervical cancer, despite its lower potency compared to conventional chemotherapy.

The IC_50_ range observed in this study is consistent with previous findings, where microencapsulated *N. lappaceum* shell extracts showed values between 1092 and 3571 µg/mL [18]. In both studies, THJ29T cells exhibited the highest IC_50_ values, suggesting an intrinsic resistance of this cell line that appears to be independent of the formulation (microencapsulated vs. non-encapsulated extract). Furthermore, the lower IC_50_ values observed in the present study, particularly in HeLa and HepG2 cells, compared with those previously reported for microencapsulated formulations, suggest differences in activity associated with the formulation tested. The high TI observed in HeLa cells reinforces the notion of tumor-selective activity, minimizing potential effects on non-tumoral cells such as NIH3T3. In contrast, previous studies evaluating *N. lappaceum* endocarp extracts obtained using different solvents (aqueous, chloroform, ethyl acetate, hexane, and methanol) reported lower IC_50_ values in HepG2 cells (63.98–183.11 µg/mL), indicating higher antiproliferative activity [9]. Similarly, strong antiproliferative activity has been described in MDA-MB-231 and MG-63 cell lines, with IC_50_ values of 12.4 µg/mL and 13.95 µg/mL, respectively [58]. These differences highlight the variability in proliferative behavior depending on extraction methods, chemical composition, and experimental models, suggesting that formulation and compound profile play a critical role in modulating biological activity.

In this regard, the antioxidant activity of the *N. lappaceum* extract, as determined by DPPH, ABTS, and TEAC assays, indicates a substantial redox capacity, although with lower efficiency compared to standard antioxidants. Because tumor cells often display a more vulnerable redox balance than non-tumoral cells, the marked antioxidant profile of the extract may be relevant to the biological effects observed, as it could contribute to alterations in cellular redox homeostasis and thereby affect key processes such as mitochondrial function, cell proliferation, and survival.

Furthermore, the chemical characterization of the extract provides a more coherent framework for interpreting these findings. The biological activity of the *N. lappaceum* extract may be attributed, at least in part, to the presence of bioactive compounds such as corilagin, geraniin, and chebulagic acid, which have been previously reported to exhibit antitumoral effects in various cancer models. In particular, the extract also contained ellagic acid-related metabolites, which may be relevant to the greater sensitivity observed in HeLa cells, given the reported susceptibility of cervical cancer models to polyphenolic compounds [59]. Corilagin has been reported to induce apoptosis and autophagy in glioma cells, associated with downregulation of NRF2 expression and modulation of apoptosis-related proteins such as Bcl-2 [60,61,62,63]. Similarly, geraniin has been shown to interfere with tumor metabolism by targeting lactate dehydrogenase A (LDHA), leading to downregulation of key enzymes involved in glycolysis and lipid biosynthesis, induction of cell cycle arrest, increased oxidative stress, and disruption of mitochondrial membrane potential [64]. In addition, chebulagic acid has demonstrated antiproliferative, pro-apoptotic, and anti-migratory effects, likely mediated through the regulation of signaling pathways such as PI3K/AKT and MAPK/ERK [65,66,67]. Taken together, these reports support the view that the antitumoral activity of the *N. lappaceum* extract is consistent with its phenolic composition, although additional studies are needed to clarify the mechanisms underlying its differential effects across tumor cell lines.

### 3.7. Hemolytic Activity

**Figure 3:**
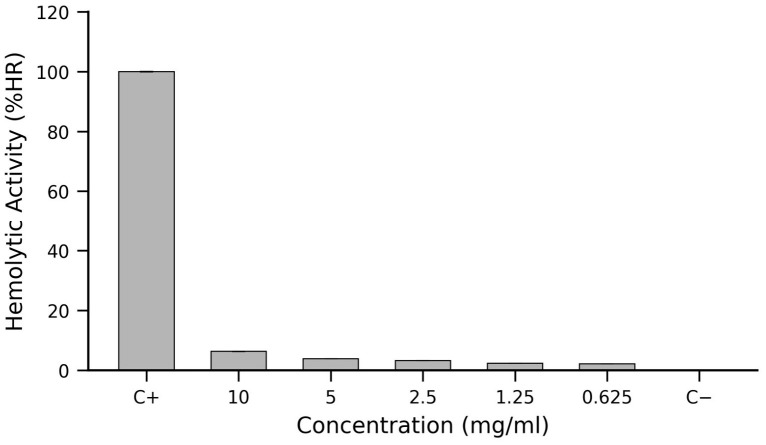
Hemolytic activity of *N. lappaceum* extract.

The *N. lappaceum* shell extract demonstrated high hemocompatibility across the evaluated concentration gradient (0.625 to 10 mg/mL). As illustrated in Figure 3, the hemolytic rate (HR) remained significantly below the 5% threshold established by the ASTM E2524-22 standard [20], except at 10 mg/mL. At concentrations ≤ 5 mg/mL, the extract can be classified as non-hemolytic, with values ranging from 3.8% to a baseline-equivalent of 2.1%.

This low hemolytic activity is particularly noteworthy given the high concentration of polyphenols identified via HPLC-MS/MS, specifically geraniin (ID4, *m*/*z* 951) and corilagin (ID3, *m*/*z* 633). While many plant extracts induce significant erythrocyte lysis due to the presence of triterpenoid saponins—which act as detergents by complexing membrane cholesterol—the absence of such compounds in the *N. lappaceum* profile fundamentally alters its biological interaction. Instead, the dominance of elagitannins suggests a “membrane-stabilizing” rather than “membrane-disrupting” mechanism. Supporting this stabilizing effect of ellagitannins, ellagitannins-enriched extract of *Punica granatum* L. protected erythrocytes membrane integrity in the presence of oxidants and restored reduced glutathione to normal levels [68].

As the extract concentration decreased from 10 to 5000 µg/mL^−1^, hemolytic activity declined accordingly. The same trend was observed at 2.5 and 1.25 mg mL^−1^, indicating a progressive reduction in membrane perturbation with dilution. At the lowest concentration tested (0.625 mg mL^−1^), the signal was only marginally above that of the negative control, suggesting that hemolytic activity at this dose was essentially negligible.

The low hemolytic activity is particularly relevant in the context of biomedical applications, as it suggests that the extract can interact with biological membranes without causing substantial cytotoxic effects. This property may be attributed to the balance between membrane-active compounds, such as saponins, and stabilizing interactions mediated by polyphenols [69,70].

Overall, the hemocompatibility profile of the extract supports its potential for further development, particularly in applications where systemic toxicity and membrane integrity are critical considerations.

## 4. Conclusions

This study demonstrates that *N. lappaceum* (rambutan) shell constitutes a highly valuable and underutilized source of bioactive compounds, characterized by a phenolic profile dominated by ellagitannins such as geraniin, corilagin, and chebulagic acid, along with ellagic acid derivatives. As previously stated, we did not identify any anthocyanins. The remarkably high total phenolic content covering several types of ellagitannins and phenolic acids, could emascarate the signal representation in HPLC-MS/MS of anthocyanins. Environmental factors (i.e., altitude, UV radiation, etc.) as well as extraction procedures could affect the anthocyanin production and recovery. Therefore, in future work we should consider these factors impact in chemical composition.

Functionally, the extract exhibited strong antioxidant capacity, with IC_50_ values indicative of high radical scavenging activity, consistent with its chemical composition. In addition, it showed broad-spectrum antibacterial activity, including effectiveness against multidrug-resistant strains, as well as significant antibiofilm properties, highlighting its potential in addressing microbial resistance and biofilm-associated infections. Importantly, the extract demonstrated selective antitumoral activity, particularly against HeLa cells, with favorable therapeutic index values, suggesting preferential cytotoxicity toward tumor cells. Furthermore, hemolytic assays confirmed a low toxicity profile, indicating good hemocompatibility and supporting its safety for potential biomedical applications.

Overall, these findings provide strong evidence that rambutan shell extract represents a multifunctional bioactive matrix with applications in pharmaceutical, nutraceutical, and food preservation fields. From a sustainability perspective, the valorization of this agro-industrial byproduct aligns with circular economy principles, transforming waste into high-value functional ingredients. Future studies should focus on elucidating molecular mechanisms of action, optimizing extraction and formulation strategies, and validating efficacy in in vivo models to facilitate its translation into practical applications.

## Figures and Tables

**Table 1:** List of antibiotics and concentrations used as controls.

Bacterial Strain	Antibiotic
*E. faecium* *	Nourseothricin (100 µg/mL)
*P. aeruginosa* *	
*K. pneumoniae* *	
*S. enterica serovar Kentucky* *	
*S. epidermidis* *	
*S. enterica* typhi ATCC 14028	Ampicillin (100 µg/mL)
*S. aureus* ATCC 25923	
*E. coli* ATCC 25922	
*L. monocytogenes* ATCC 13932	
*P. aeruginosa* ATCC 27853	Tetracycline (10 µg/mL)
*E. faecalis* ATCC 29212	

* Clinical multidrug-resistant isolates.

**Table 2:** Average levels of bioactive compounds of the *N. lappaceum* shell.

Parameter	Shell Extract		
Total anthocyanin (mg D-ch/100 g DW)	17.63	±	4.60
Vitamin C (mg/100 g DW)	1.15	±	0.1
Phenolics (mg/100 g DW)			
Gallic acid	250.6	±	3.3
Vanillic acid	67.6	±	1.4
Protocatechic acid	2414.4	±	71.0
m-Coumaric acid	21,471.3	±	239.4
Syringic acid	1331.7	±	7.5
Kaempferol	446.7	±	58.2
Total phenolics	25,982.2	±	380.8
Organic acid (mg/g DW)			
Tartaric acid	37.9	±	3.6
Malic acid	488.1	±	7.5
Citric acid	230.7	±	23.7
Total organic acid	756.8	±	10.3

D-ch, Delphinidin chloride; DW, dry weight.

**Table 4:** *N. lappaceum* extract antioxidant activity measured by DPPH, ABTS and TEAC methods.

Compound	DPPH IC_50_ (μg/mL)	ABTS IC_50_ (μg/mL)	TEAC * (μmol TE/g)
*N.lappaceum* extract	8.02 ± 0.94	89.78 ± 11.82	5703.92 ± 304.92
Ascorbic acid	1.38 ± 0.19	57.58 ± 16.33	10,327.59 ± 3461.55

* TEAC based on the ABTS assay only.

**Table 5:** Minimal inhibitory concentration (MIC) for different bacterial strains.

Bacterial Strain	MIC (mg/mL)
*Salmonella enterica typhi* ATCC 14028	10
*Escherichia coli* ATCC 25922	10
*Staphylococcus aureus* ATCC 25923	2.5
*Listeria monocytogenes* ATCC 13932	5
*Enterococcus faecalis* ATCC 29212	10
*Pseudomonas aeruginosa* ATCC 27853	10
*Klebsiella pneumoniae* *	20
*Escherichia coli* *	20
*Enterococcus faecalis* *	10
*Staphylococcus epidermidis* *	10
*Enterococcus faecium* *	10
*Salmonella enterica serovariedad Kentucky* *	10
*Pseudomonas aeruginosa* *	20

* Clinical multidrug-resistant isolates.

**Table 6:** Half-maximal inhibitory concentration values (IC_50_) (μg/mL) against tumor and non-tumor cell lines at 72 h and therapeutic index (TI) values. Values are expressed as mean ± standard deviation, *n* = 4. TI values were calculated as IC_50_ (NIH3T3)/IC_50_ (tumor cell). ^b^ Therapeutic index (TI) was calculated as the ratio of IC_50_ in NIH3T3 (non-tumor) cells to IC_50_ in the respective tumor cell line: TI = IC_50_ (NIH3T3)/IC_50_ (tumor cell line).

	THJ29T		HepG2		HCT116		HeLa		NIH3T3
	IC_50_	TI ^b^	IC_50_	TI ^b^	IC_50_	TI ^b^	IC_50_	TI ^b^	IC_50_
Extract	3086 ± 0.4	0.9	836 ± 0.1	3.4	1059 ± 0.2	2.7	260 ± 0.0	11.5	2678 ± 0.6
Cisplatin	4.6 ± 0.5	0.6	3.7± 0.4	0.7	2.3 ± 0.4	1.2	1.2 ± 0.1	2.3	2.7± 0.5

## Data Availability

The original data presented in the study are openly available in FigShare at https://doi.org/10.6084/m9.figshare.32086794 (accessed on 23 April 2026).
